# Chokeberry Pomace as a Determinant of Antioxidant Parameters Assayed in Blood and Liver Tissue of Polish Merino and Wrzosówka Lambs

**DOI:** 10.3390/molecules22111461

**Published:** 2017-11-07

**Authors:** Paulina Lipińska, Atanas G. Atanasov, Marek Palka, Artur Jóźwik

**Affiliations:** 1 Institute of Genetics and Animal Breeding of the Polish Academy of Sciences, 05-552 Jastrzebiec, Poland; a.atanasov.mailbox@gmail.com (A.G.A.); aa.jozwik@ighz.pl (A.J.); 2 Department of Pharmacognosy, University of Vienna, 1090 Vienna, Austria; 3 Faculty of Power and Aeronautical Engineering, Warsaw University of Technology, 00-665 Warsaw, Poland; marek.palka@int.pl

**Keywords:** nutraceuticals, oxidative stress, antioxidative enzymes, antioxidative peptides, lipid peroxidation, phenolic compounds

## Abstract

Despite being a plant by-product, chokeberry pomace is believed to exert some therapeutic effects because it is one of the richest sources of highly bioavailable non-enzymatic antioxidants. The aim of this study was to determine the functionality of bioactive compounds present in the *Aronia melanocarpa* pomace (chokeberry) based on enzymatic and non-enzymatic parameters related to the active defence of liver and blood against the effects of oxidative stress. The experiment was conducted with 48 lambs of two breeds—Polish Merino and Wrzosówka. Experimental groups were administered the basic feed with the addition of 150 g or 300 g of black chokeberry pomace per each kg of the complete feed. The activities of antioxidative enzymes (superoxide dismutase, glutathione peroxidase), peptides (glutathione, glutathione disulfide), and a lipid peroxidation indicator (malondialdehyde), as well as the capacity of non-enzymatic antioxidants were investigated. The results proved a strong effect of bioactive compounds contained in the black chokeberry pomace on the estimated parameters. The inclusion of chokeberry pomace in feed mixtures brought many benefits linked with the antioxidative protection. Parameters responsible for the oxidative status were significantly modified despite the commonly-held view about a limited possibility of transferring phenolic compounds to the organs.

## 1. Introduction

Biological mechanisms that protect aerobic organisms against reactive oxygen species include the activities of specialized enzymatic proteins and peptides [1,2,3,4,5]. A glutathione molecule is highly reactive due to its characteristic structure [6,7,8]. Superoxide dismutases are significant elements of cell defence against the toxic effects of free radicals [9]. Glutathione peroxidase is present mostly in the cytosol, followed by mitochondria and cell nucleus, and its highest activity is reported in the liver, which is associated with detoxification processes proceeding in this organ [10]. Antioxidants prevent lipid peroxidation (malondialdehyde is often assayed as an indicator of this process) and actively control its course [11]. Determination of the total antioxidative potential of bioactive compounds and the potential of scavenging free radicals is very useful in neutralizing the reactivity of free radicals [12], and thus the malondialdehyde concentration [13]. Plants are an important source of diverse compounds with health-promoting bioeffects [14,15,16]. *Aronia melanocarpa* (chokeberry) is known as a rich source of compounds with strong antioxidative properties [17]. Due to their health-promoting properties, phenolic compounds constitute the most important bioactive components of its fruit [12,17,18]. Chokeberry pomace is much richer in compounds with a strong antioxidative potential than the food products made of its fruit [17,19,20]. Polymeric proanthocyanins are the major class of polyphenolic compounds in chokeberry, representing 66% of the fruit’s polyphenols. The average concentration ranged from 1578.79 mg/100 g for chokeberry juice up to 8191.58 mg/100 g in pomace [17]. Anthocyanins in pomace (made form *A. melanocarpa*) are a second phenolic compound group, and represent about 25% of total polyphenols, a mixture of four different cyanidin glycosides: 3-galactoside, 3-glucoside, 3-arabinoside, and 3-xyloside [17]. 

The aim of this study was to establish the impact of chokeberry pomace addition to a lamb diet on parameters indicating the active defence of cells against effects of oxidative stress. Stimulation of enzymatic proteins and peptides by compounds with strong antioxidative properties enables evaluation of the effects resulting from health-improving properties of phenolic compounds. The perspective of the multi-directional use of chokeberry pomace may contribute to the introduction of new trends in the feeding of farm animals.

## 2. Results

### 2.1. Activity of Superoxide Dismutase (SOD) and Glutathione Peroxidase (GPx)

The application of chokeberry pomace in the diets of Polish Merino and Wrzosówka lambs resulted in interesting changes in the activity of superoxide dismutase. The activity of SOD in serum (Table 1) was higher in the Polish Merino control group (C_MP_) than in the experimental group (Ex_PM150_, Ex_PM300_). However, there were no differences between Ex_PM150_ and Ex_PM300_ groups.

Both experimental groups from the Polish Merino breed responded in the same way, regardless of the amount of additive. In the case of the Wrzosówka breed, a reduction of SOD activity was observed only in the experimental group fed with the addition of 300 g of chokeberry pomace (Table 1). Control group and Ex_W150_ were similar. A lower level of SOD activity in C_W_ than C_PM_ was observed, as well as in Ex_W300_ compered to Ex_PM300_ (Table 2).

Moreover, the results of our study demonstrated a significant increase of SOD activity in the liver of lambs from the groups Ex_PM150_, Ex_PM300_, Ex_W150_, and Ex_W300_ as compared to control animals (Table 1). Besides, the C_MP_ group had lower activity of liver SOD than C_W_ (Table 2).

The addition of chokeberry pomace stimulated the activity of glutathione peroxidase as well. GPx activity was lower in plasma from C_PM_ and C_W_ groups compared to Ex_PM150_, Ex_PM300_, Ex_W150_, and Ex_W300_ (Table 3).

There were no differences between breeds (Table 2). The GPx activity in the liver was significantly lower in lambs from the Ex_PM300_ group compared to those from C_PM_ and Ex_PM150_ groups, which were similar to each other. Additionally, Ex_PM300_ had lower activity than Ex_W300_ (Table 2).

The obtained results are especially interesting from the viewpoint of the functions and metabolism of non-enzymatic antioxidative compounds—in particular, the tissues of lambs of both breeds and their effect on antioxidative enzymes. Interestingly, the deposition of enzymatic antioxidants is not the same in liver and serum.

### 2.2. Reduced Glutathione (GSH) Level

In the experimental groups, a significant increase was determined in a reduced glutathione (GSH) level in the blood of lambs (Table 4 and Table 5). The highest level of GSH was observed in the groups Ex_PM300_ and Ex_W300_. Oxidized glutathione (GSSG) was below the limit of quantification of the applied test (<0.001 µm).

As it is known, glutathione exists in both reduced (GSH) and oxidized (GSSG) states. In the reduced state, the thiol group of cysteine is able to donate a reducing equivalent to other molecules (e.g., reactive oxygen species) to neutralize them, or to protein cysteines to maintain their reduced forms. It may be thought that GSSG level below the limit of quantification was caused by a low amount of free radicals, which may initiate GSH oxidation.

### 2.3. The Level of Malondialdehyde (MDA)

The results obtained demonstrate a considerable decrease in the MDA level in the liver of lambs from groups Ex_PM150_, Ex_PM300_, Ex_W150_, and Ex_W300_ (Table 6). Significant differences between the breeds were observed as well: Ex_PM300_ and Ex_W300_; Ex_PM150_ and Ex_W150_ (Table 5). In the blood samples, the MDA level was below the limit of quantification of the applied test. This may be caused by a high level of non-enzymatic antioxidants in examined samples which effectively protect the cell against lipid peroxidation.

### 2.4. The Level of Non-Enzymatic Antioxidant Capacity (NEAC)

In our study, a significant increase in NEAC was observed in the experimental groups as compared to the control, depending on the amount of additive. The liver showed a greater responsiveness to the applied experimental factor as compared to the serum (Table 7). The breed of animals had no significant effect on this parameter (Table 5).

## 3. Discussion

The amount of the additive had a significant impact on most of the studied parameters. Nevertheless, differences between investigated indicators are not as obvious as was expected. A double content of the additive did not translate into a double effect. Differences were observed depending on the breed and type of tissue as well. Obtained results show that antioxidative enzymes, peptides, and oxidative status indicators may respond to chokeberry pomace addition in various ways, as described in detail below. Antioxidative defence of aerobic organisms may be more complex and difficult to modulate than previously thought.

Studies on superoxide dismutase bring information on this subject which do not always indicate an identical cause of the reduction or increase in its activity. Results obtained in this study are different in comparison to the work in which the authors interpret the increase in activity of SOD in the serum of lambs as an effect of feed with the supplement of purple corn pigment [21]. Additionally, in the other research the application of natural supplements rich in antioxidants (tomato pomace, grape skin extract) resulted in the increase in activity of SOD in the blood of Bergamasca sheep [22]. However, according to this article, the animals were exposed to hand-on stress induced by handling [22]. Therefore, this could have been the main reason for the increase in the SOD activity in the obtained samples. The diversity of biologically-active compounds present in natural products of vegetable origin means that the application of a similar experimental factor does not always render identical results. However, the increase in SOD activity in the liver of the lambs from the experimental groups and in the serum of lambs of the Suffolk breed [21] could have been due to the feed additive applied. In another study [23], a significant impact of purple potato flakes on the activity of SOD was reported in rat livers. Similar correlations were obtained in research on the effect of sour cherry juice on SOD activity in mice liver [24]. Results obtained confirm that the increase in SOD activity in the liver was related to the properties of hepatic phenolics [25,26,27]. 

In comparable studies [28], an increase in the activity of anti-oxidative enzymes (including GPx) is correlated with the induction of oxidative stress. In addition, the use of a supplement with anti-oxidative properties (tomato pulp, grape peel, vitamin E) also caused this effect [22]. Therefore, in spite of obtaining similar tendencies, it should be pointed out that they resulted from different factors. The activation of glutathione peroxidase in the serum occurred as a result of the functional nature of phenol compounds, which constitute a perfect defence against oxidative stress [21,29]. A decrease in GPx activity in the liver may be due to a high activity of SOD in this organ. The reaction of glutathione oxidation—the intensity of which depends on the activity of GPx—takes place under conditions of oxidative stress. It may therefore be hypothesized that superoxide dismutase takes over the first line of defence against free radicals, which results in GPx activity decrease. Similar observations were made for blood samples, where—as a result of dietary inclusion of the additive—the activity of GPx increased while SOD activity remained unchanged. 

Glutathione level measurements are very useful in evaluating the effectiveness of the intervention strategy of antioxidants. Nevertheless, results obtained by other works [21,30] present no impact of a supplement rich in phenol compounds on the glutathione level in the blood of lambs. It may be interpreted as phenomenon referring to the increased activity of SOD in the blood of experimental groups of animals [30]. In our study, no increase in the SOD activity was observed in the serum of lambs upon the use of chokeberry pomace. However, glutathione showed a specific response to attempts of stimulating its level. Therefore, the observed increase in the level of reduced glutathione in the blood may result from the specific properties of compounds of antioxidative nature present in the applied additive.

The oxidative degradation of lipids is the process in which free radicals gather electrons from the lipids in cell membranes, resulting in cell damage. The end products of lipid peroxidation are reactive aldehydes, such as malondialdehyde (MDA). It seems that a lower level of MDA in examined samples is correlated with a higher level of non-enzymatic antioxidants which neutralize free radicals and protect the cell membrane. The obtained results prove that the decrease in the MDA level was strictly connected with the additive amount. Additionally, in other studies the MDA level was reduced after the application of antioxidative plant additive [22,27,31]. Moreover, a substantial impact of stress stimulus on the increase in MDA level, which was ultimately reduced owing to the application of plant compounds with antioxidative properties, was noticed [21].

Many studies have confirmed that chokeberry pomace has the highest content of NEAC among the berry by-products [32,33]. During the experiment, it was observed that the application of chokeberry pomace resulted in an increase of the non-enzymatic total antioxidant capacity (NEAC) in all experimental groups [34,35]. However, recent studies focused on this subject were conducted on monogastric organisms. The specific nature of the digestive system of ruminants requires referencing the results to the work based on this group of animals. However, in a similar experiment, authors did not observe NEAC increase in the serum of the Suffolk sheep, despite application of the supplement rich in phenol compounds [21]. The lack of increase in the NEAC and glutathione level, with a simultaneous increase in SOD activity in this research [21], does not give answers concerning the usability of the supplement in counteracting oxidative stress factors.

## 4. Materials and Methods

### 4.1. Livestock and Diet

The experiment was conducted with 48 lambs of two breeds—the Polish Merino (PM) and the Wrzosówka (W). Since birth, the ram lambs were kept under the same conditions for 60 days, and then were separated from the ewes and randomly placed in group pens. Animals of both breeds were divided into three dietary groups: control group (C_PM_; C_W_) and two experimental groups differing in chokeberry pomace addition to diet (Ex_PM150_; Ex_PM300_; Ex_W150_; Ex_W300_). Each group included eight lambs of two breeds. They were kept for 90 days in conditions of an experimental farm with full welfare. All groups received compound feed, balanced in accordance with dietary standards for lambs aged 60 to 150 days. Apart from the basic feed, lambs from the experimental groups received an additive in the form of 150 g (Ex_PM150_; Ex_W150_) or 300 g (Ex_PM300;_ Ex_W300_) of chokeberry pomace per each kg of the complete compound feed. Chemical composition of all applied feed was analysed (Table 8 and Table 9). Chemical composition of chokeberry pomace has a very low level of protein or fat. That is why it has no influence on a balanced diet. 

Chokeberry pomace might be treated as a normal component of feed for the animals, like other by-products commonly applied in livestock fattening. It ensures a health-promoting effect without the risk of adverse interference with the metabolism of animals. However, The main reasons for choosing chokeberry pomace as a feed additive were its unique antioxidative properties. Therefore, nonenzymatic antioxidants capacity in all types of feed was investigated as well (Table 9).

Chokeberry pomace is much richer in compounds with a strong autoxidation potential than other food by-products. All of its valuable components are not lost during processing because they occur mainly in the skin of the chokeberry fruit. The bioactive impact of compounds present in *A. melanocarpa* pomace on small ruminants has not been thoroughly investigated. There is a huge variety of plant antioxidants with extremely different metabolism. However, the bioavailability of chokeberry pomace antioxidants is absolutely unique and could not be compared with other plant additives used in livestock feeding, as has been done in other studies. Lambs were chosen as an animal model because of their ability to digest and absorb bioactive compounds from plant industry by-products such as chokeberry pomace. 

The energy and protein values of all feeds were determined on the basis of the results of chemical analyses. This allowed balancing contents of energy, proteins, and fibres in the three types of diets in accordance with the INRA standards. Lambs received the feed twice a day. The quantity of feed was monitored and adjusted to the fattening period. 

The animals were slaughtered at the age of 150 days in a certified slaughterhouse located within the Experimental Department of Institute of Genetics and Animal Breeding of the Polish Academy of Sciences. The experimental farm possesses all necessary permits and licenses to work with animals. Care was taken to prevent the animals from excessive physical activity and nervous system stimulation before slaughter. The lambs were fasted 24 h before slaughter (they received only fresh water). They were delivered to the slaughterhouse individually. During the slaughter, the lambs were fully unconscious. Animals shall only be killed after stunning in accordance with the methods and specific requirements related to the application of those methods concluded in the European Union Council Regulation No. 1099/2009. Lambs were stunned with the use of a penetrative captive bolt device as recommended in annex No. 1 of the European Union Council Regulation No. 1099/2009 (Slaughter of small ruminants). All operations were conducted in accordance with guidelines of the Chief Veterinary Inspectorate, in the presence of a Poviat (County) veterinary officer. According to relevant national guidelines, The Second Local Ethics Committee for Experiments on Animals at the Warsaw University of Life Sciences made a declaration (before the experiment was started) that the study did not require any special permission, which was confirmed by an official statement—a respective Ethics Committee provided a waiver of the need for ethical approval for the conducted study. No factors that could cause animal suffering were used during the study. All animals involved in the experiment were subject to farm slaughter.

Blood was collected after stunning by insertion of a needle into the jugular vein. Samples of liver tissue were collected immediately after slaughter by biopsy of the tissue from the entire organ. All blood and liver tissue samples were collected by a qualified veterinary doctor who had all necessary permits to work in a slaughterhouse. 

### 4.2. Superoxide Dismutase Activity

Liver samples were perfused with a PBS buffer at pH 7.4. The tissue was homogenised in 5 mL of 20 mM HEPES buffer with pH 7.2 and chilled to 4 °C, with addition of 1 mM EDTA (PubChem CID:8759), 210 mM mannitol (PubChem CID:6251), and 70 mM sucrose (PubChem CID:5988) per 1 g of tissue. The homogenates were centrifuged at the speed of 1.500× *g* for 5 min at a temperature of 4 °C. In order to assay the activity of superoxide dismutase (SOD) in blood, the samples were collected into individual sterile test tubes without anticoagulant. The serum was obtained by sample centrifugation at 2.500× *g* for 15 min at 4 °C. All samples were stored in ice until the analysis. The SOD analysis was conducted using the Cayman Chemical Company test (Ann Arbor, MI 48108, USA), and enabled the collection of data on all three types of SOD (Cu/Zn, Mn, FeSOD). The reactions occurring during the test are based on the use of a tetrazolium salt to detect reactive oxygen species generated by xanthine and hypoxanthine. Absorbance was read at three wavelengths (λ_440, 450, 460_) using a Synergy4 microplate reader (Biotek; Winooski, VT, USA). Calculations of SOD activity were made with Gen5 software (Biotek; Winooski, VT, USA). The activity of SOD was expressed in U/mL.

### 4.3. Glutathione Peroxidase Activity

Liver samples were perfused with a PBS buffer at pH 7.4. The tissue was homogenised in 5 mL of the buffer, chilled to 4 °C, consisting of 50 mM Tris-HCL, 5 mM EDTA, and 1 mM dithiothreitol (PubChem CID:19001). The samples were centrifuged at 10,000× *g* for 15 min at 4 °C. The resultant supernatant was stored on ice until the analysis. In order to assay the activity of glutathione peroxidase (GPx) in plasma, the material was collected in individual sterile test tubes containing ammonium heparin. The samples were centrifuged at 1000× *g* for 10 min at 4 °C. The activity of GPx was determined using the Cayman Chemical Company test (Ann Arbor, MI, USA). The reaction of NADPH (PubChem CID:5884) oxidation to NADP^+^ enables the detection of changes in absorbance (λ_340_). Absorbance readings and the measurement of reaction kinetics were performed using a Synergy4 microplate reader (Biotek; Winooski, VT, USA). The results were calculated using Gen5 software (Biotek, Winooski, VT, USA). The activity of GPx was expressed in nmol/min/mL. 

### 4.4. Glutathione, Glutathione Disulphide Content

Glutathione concentration was determined in the full blood, which required collecting the material in test tubes with an anticoagulant (EDTA). In the full blood tests, glutathione was determined by means of the OxisReasearch™ Bioxytech^®^ GSH/GSSG—412™ test (Foster City, CA, USA). Before the analysis, the samples were frozen with the addition of M2VP (1-methyl-2-vinyl-pyridium trifluoromethane sulphonate) at a temperature of −80 °C, and then thawed in order to initiate the erythrosis process. The released reduced glutathione (GSH) and oxidised glutathione (GSSG) were determined in accordance with the detailed instruction provided by the kit’s producer. Absorbance reading (λ_412_) and the measurement of reaction kinetics were performed using a microplate reader Synergy4 (Biotek; Winooski, VT, USA). The results were calculated using Gen5 software (Biotek). Glutathione concentration was expressed in µM.

### 4.5. Malondialdehyde Concentration

Liver tissue was perfused with a phosphate buffer at pH 7.4. Then, the tissue was homogenised in 2 mL of a phosphate buffer, chilled to 4 °C, with the addition of 20 µL of butylated hydroxytoluene (PubChem CID:31404) in acetonitrile. The samples were centrifuged at 10,000× *g* for 10 min at 4 °C. The resultant supernatant was stored on ice until the analysis. Determination of MDA level in blood required collecting the material in test tubes containing ammonium heparin. The samples were centrifuged at 1000× *g* for 10 min at 4 °C. Further procedures followed strict instructions of the producer of the OxisReasearch™ Bioxytech^®^ MDA-586™ test (Foster City, CA, USA). The test is based on the reaction of *N*-methyl-2-phenylindole (NMPI) with MDA at a temperature of 45 °C. One MDA molecule reacts with two NMPI molecules, creating a stable, coloured complex. The OxisReasearch™ Bioxytech^®^ MDA-586™ test is precise, and conditions of its use eliminate the risk of interference with other products of lipid peroxidation. The absorbance reading (λ_586_) was made using a Cary Varian 50Bio spectrometer (Santa Clara, CA, USA). Calculations were performed on the basis of a calibration curve obtained according to the producer’s recommendations and the template included in the test’s report. The concentration of MDA was expressed in µM.

### 4.6. Non-Enzymatic Total Antioxidant Capacity

The biological material (liver tissue, serum) was processed according to the instructions of the Total Antioxidant Capacity Assay Kit by Abnova^®^ (Taoyuan City, Taiwan), strictly following the manufacturer’s recommendations. This test does not require sample purification. The tissue was homogenised in 1.96 mL of demineralised water (ddH_2_O), chilled to 4 °C, with the addition of 40 µL of dimethyl sulphoxide (PubChem CID:679). The method is based on the measurement of reduction of indicator ions, which as a result of reaction with the sample produce a coloured complex. Absorbance was measured at λ_570_. The total antioxidant capacity was expressed in mM, per single—electron Trolox equivalent. 

### 4.7. Statistical Analysis

The results were subjected to statistical treatment with the use of statistical software IBM SPSS 18 (IBM Corporation, Foster City, CA, USA) using a general linear model (GLM). The conducted multifactorial analysis of variance (breed, feeding) ANOVA enabled the identification of significant effects of differences at the level of *p* ≤ 0.01. The parameters of dependent variables were measured on a quantitative scale, and their distributions were similar to normal distributions. Statistical corrections (Pillai trace, Wilks’ lambda, Hotelling’s trace, largest Roy’s element) were applied. The multiple pairwise comparisons test and the performed post hoc Bonferroni procedure showed that there were significant differences between the averages for each pair of groups.

## 5. Conclusions

The most important biochemical markers were examined in this study, which are directly related to the cascade of oxidative stress processes. The obtained results indicate the health-promoting effect of the chokeberry pomace on lamb organisms, which might be caused by the high bioavailability of polyphenolic compounds that are contained in the supplied additive. The inclusion of chokeberry pomace in feed mixtures for lambs of Polish Merino and Wrzosówka breeds brought many benefits linked with antioxidative protection. Parameters responsible for the oxidative status of expatiated tissue were significantly modified, despite a commonly-held view about there being no possibility of transferring phenolic compounds to the organs. In the authors’ opinion, the observed effects are caused by unique properties of chokeberry pomace, the bioavailability of their antioxidative compounds, and characteristic traits of the gastrointestinal tract of small ruminants. Animal production is based on a well-balanced diet, welfare, and good health of the livestock. Reduction of oxidative stress is crucial in animal production (especially in liver and blood). A properly functioning liver, without hepatic steatosis, equipped with active defence of antioxidative enzymes, protein, and polyphenols allow young animals to grow in a more beneficial way. This research provides a novel insight into the perception of polyphenolic compound metabolism in ruminant organisms and may pave the way for further investigations addressing this research problem.

## Figures and Tables

**Table 1:** Activity of superoxide dismutase (U/mL) in serum and liver samples collected from both breeds as affected by the dose of the additive.

Type of Sample	Breed	Mean			Statistical Significance	Standard Deviation
Serum	Polish Merino	Ex_PM150_	5.921 U/mL	Ex_PM300_	1.000	±0.714
				C_PM_	0.000 **	
		Ex_PM300_	5.882 U/mL	Ex_PM150_	1.000	±0.978
				C_PM_	0.000 **	
		C_PM_	8.591 U/mL	Ex_PM150_	0.000 **	±1.150
				Ex_PM300_	0.000 **	
	Wrzosówka	Ex_W150_	5.651 U/mL	Ex_W300_	0.003 **	±0.969
				C_W_	1.000	
		Ex_W300_	3.803 U/mL	Ex_W150_	0.003 **	±1.117
				C_W_	0.001 **	
		C_W_	5.788 U/mL	Ex_W150_	1.000	±1.198
				Ex_W300_	0.001 **	
Liver	Polish Merino	Ex_PM150_	34.033 U/mL	Ex_PM300_	0.791	±2.776
				C_PM_	0.000 **	
		Ex_PM300_	36.438 U/mL	Ex_PM150_	0.791	±4.120
				C_PM_	0.000 **	
		C_PM_	10.176 U/mL	Ex_PM150_	0.000 **	±2.543
				Ex_PM300_	0.000 **	
	Wrzosówka	Ex_W150_	33.935 U/mL	Ex_W300_	0.305	±6.473
				C_W_	0.000 **	
		Ex_W300_	37.488 U/mL	Ex_W150_	0.305	±5.517
				C_W_	0.000 **	
		C_W_	15.343 U/mL	Ex_W150_	0.000 **	±2.146
				Ex_W300_	0.000 **	

The multiple pairwise comparisons test and post hoc Bonferroni procedure showed some significant differences between the averages for each pair of groups: * means *p* ≤ 0.05, ** means *p* ≤ 0.01. C_PM,_ C_W_—control group, Experimental groups—Ex_PM150_, Ex_PM300_, and Ex_W150_, Ex_W300_ (150 or 300 g of chokeberry pomace additive per each kg of the complete compound feed).

**Table 2:** Differences between the antioxidative enzyme activity of each pair of breeds.

Type of Sample and Enzyme	Amount of Additive	Polish Merino compared to Wrzosówka		
		Statistical Significance	Difference between Averages	Standard Error
Serum SOD	150 g	0.603	±0.271	0.517
	300 g	0.000 **	±2.079	0.517
	control	0.000 **	±2.803	0.517
Liver SOD	150 g	0.963	±0.098	2.122
	300 g	0.624	±1.049	2.122
	control	0.019 *	±5.168	2.122
Plasma GPx	150 g	0.259	±14.375	12.553
	300 g	0.325	±12.500	12.553
	control	0.692	±5.000	12.553
Liver GPx	150 g	0.124	±9.125	5.812
	300 g	0.008 **	±16.057	5.812
	control	0.308	±6.000	5.812

The multiple pairwise comparisons test and post hoc Bonferroni procedure showed some significant differences between antioxidative enzymes activity of each pair of breeds: * means *p* ≤ 0.05, ** means *p* ≤ 0.01. GPx: glutathione peroxidase; SOD: superoxide dismutase.

**Table 3:** Activity of glutathione peroxidase (nmol/min/mL) in plasma and liver samples collected from both breeds as affected by the dose of the additive.

Type of Sample	Breed	Mean			Statistical Significance	Standard Deviation
Plasma	Polish Merino	Ex_PM150_	233.375 nmol/min/mL	Ex_PM300_	0.056	±23.766
				C_PM_	0.001 **	
		Ex_PM300_	264.125 nmol/min/mL	Ex_PM150_	0.056	±19.276
				C_PM_	0.000 **	
		C_PM_	183.750 nmol/min/mL	Ex_PM150_	0.001 **	±23.927
				Ex_PM300_	0.000 **	
	Wrzosówka	Ex_W150_	247.750 nmol/min/mL	Ex_W300_	0.079	±34.400
				C_W_	0.000 **	
		Ex_W300_	276.625 nmol/min/mL	Ex_W150_	0.079	±27.076
				C_W_	0.000 **	
		C_W_	188.750 nmol/min/mL	Ex_W150_	0.000 **	±18.889
				Ex_W300_	0.000 **	
Liver	Polish Merino	Ex_PM150_	56.125 nmol/min/mL	Ex_PM300_	0.030 *	±9.613
				C_PM_	0.548	
		Ex_PM300_	40.443 nmol/min/mL	Ex_PM150_	0.030 *	±8.596
				C_PM_	0.001 **	
		C_PM_	64.000 nmol/min/mL	Ex_PM150_	0.548	±13.564
				Ex_PM300_	0.001 **	
	Wrzosówka	Ex_W150_	65.250 nmol/min/mL	Ex_W300_	0.419	±9.377
				C_W_	1.000	
		Ex_W300_	56.500 nmol/min/mL	Ex_W150_	0.419	±7.760
				C_W_	0.075	
		C_W_	70.000 nmol/min/mL	Ex_W150_	1.000	±7.559
				Ex_W300_	0.075	

The multiple pairwise comparisons test and post hoc Bonferroni procedure showed significant differences between the averages for each pair of groups: * means *p* ≤ 0.05, ** means *p* ≤ 0.01. C_PM,_ C_W_—control group, Experimental groups—Ex_PM150_, Ex_PM300_ and Ex_W150_, Ex_W300_ (150 or 300 g of chokeberry pomace additive per each kg of the complete compound feed).

**Table 4:** Capacity of reduced glutathione (µM) in full blood collected from both breeds as affected by the dose of the additive.

Type of Sample	Breed	Mean			Statistical Significance	Standard Deviation
Full blood	Polish Merino	Ex_PM150_	357.625 µM	Ex_PM300_	0.003 **	±35.230
				C_PM_	0.005 **	
		Ex_PM300_	556.625 µM	Ex_PM150_	0.002 **	±76.052
				C_PM_	0.008 **	
		C_PM_	267.875 µM	Ex_PM150_	0.005 **	±39.244
				Ex_PM300_	0.008 **	
	Wrzosówka	Ex_W150_	408.500 µM	Ex_W300_	0.008 **	±59.809
				C_W_	0.007 **	
		Ex_W300_	544.375 µM	Ex_W150_	0.003 **	±63.895
				C_W_	0.006 **	
		C_W_	263.375 µM	Ex_W150_	0.006 **	±31.122
				Ex_W300_	0.006 **	

The multiple pairwise comparisons test and post hoc Bonferroni procedure showed significant differences between the averages for each pair of groups: * means *p* ≤ 0.05, ** means *p* ≤ 0.01. C_PM,_ C_W_—control group, Experimental groups—Ex_PM150_, Ex_PM300_ and Ex_W150_, Ex_W300_ (150 or 300 g of chokeberry pomace additive per each kg of the complete compound feed).

**Table 5:** Differences between the level of oxidative status indicators for each pair of breeds.

Type of Sample and Enzyme	Amount of Additive	Polish Merino compared to Wrzosówka		
		Statistical Significance	Difference between Averages	Standard Error
Full blood GSH	150 g	0.064	±50.875	18.926
	300 g	0.650	±12.250	18.926
	control	0.867	±4.500	18.926
Liver MDA	150 g	0.000 **	±7.625	1.316
	300 g	0.020 *	±4.500	1.316
	control	0.088	±3.250	1.316
Serum NEAC	150 g	0.582	±0.864	1.100
	300 g	0.041 *	±3.277	1.100
	control	0.463	±1.153	1.100
Liver NEAC	150 g	0.825	±0.293	0.931
	300 g	0.272	±1.467	0.931
	control	0.930	±0.117	0.931

The multiple pairwise comparisons test and post hoc Bonferroni procedure showed some significant differences between the level of oxidative status indicators for each pair of breeds: * means *p* ≤ 0.05, ** means *p* ≤ 0.01. MDA: malondialdehyde; NEAC: non-enzymatic antioxidant capacity.

**Table 6:** The level of malondialdehyde (µM) in liver as affected by the dose of the additive.

Type of Sample	Breed	Mean			Statistical Significance	Standard Deviation
Liver	Polish Merino	Ex_PM150_	28.500 µM	Ex_PM300_	0.000 **	±4.175
				C_PM_	0.139	
		Ex_PM300_	17.250 µM	Ex_PM150_	0.000 **	±4.234
				C_PM_	0.000 **	
		C_PM_	32.125 µM	Ex_PM150_	0.174	±4.390
				Ex_PM300_	0.000 **	
	Wrzosówka	Ex_W150_	20.875 µM	Ex_W300_	0.000 **	±3.563
				C_W_	0.000 **	
		Ex_W300_	12.750 µM	Ex_W150_	0.000 **	±1.832
				C_W_	0.000 **	
		C_W_	28.875 µM	Ex_W150_	0.000 **	±3.523
				Ex_W300_	0.000 **	

The multiple pairwise comparisons test and post hoc Bonferroni procedure showed significant differences between the averages for each pair of groups: * means *p* ≤ 0.05, ** means *p* ≤ 0.01. C_PM,_ C_W_—control group, Experimental groups—Ex_PM150_, Ex_PM300_ and Ex_W150_, Ex_W300_ (150 or 300 g of chokeberry pomace additive per each kg of the complete compound feed).

**Table 7:** Non-enzymatic total antioxidant capacity (mM/single-electron Trolox equivalent) in serum and liver as affected by the dose of the additive.

Type of Sample	Breed	Mean			Statistical Significance	Standard Deviation
Plasma	Polish Merino	Ex_PM150_	19.046 mM/single-electron Trolox equivalent	Ex_PM300_	0.007 **	±2.960
				C_PM_	0.026 *	
		Ex_PM300_	24.123 mM/single-electron Trolox equivalent	Ex_PM150_	0.007 **	±2.795
				C_PM_	0.000 **	
		C_PM_	14.751 mM/single-electron Trolox equivalent	Ex_PM150_	0.026 *	±3.086
				Ex_PM300_	0.000 **	
	Wrzosówka	Ex_W150_	19.910 mM/single-electron Trolox equivalent	Ex_W300_	0.000 **	±4.993
				C_W_	0.041 *	
		Ex_W300_	27.400 mM/single-electron Trolox equivalent	Ex_W150_	0.000 **	±2.298
				C_W_	0.000 **	
		C_W_	15.904 mM/single-electron Trolox equivalent	Ex_W150_	0.041 *	±1.530
				Ex_W300_	0.000 **	
Liver	Polish Merino	Ex_PM150_	24.997 mM/single-electron Trolox equivalent	Ex_PM300_	0.000 **	±1.874
				C_PM_	0.000 **	
		Ex_PM300_	36.293 mM/single-electron Trolox equivalent	Ex_PM150_	0.000 **	±1.486
				C_PM_	0.000 **	
		C_PM_	12.923 mM/single-electron Trolox equivalent	Ex_PM150_	0.000 **	±0.674
				Ex_PM300_	0.000 **	
	Wrzosówka	Ex_W150_	25.290 mM/single-electron Trolox equivalent	Ex_W300_	0.000 **	±2.016
				C_W_	0.000 **	
		Ex_W300_	34.826 mM/single-electron Trolox equivalent	Ex_W150_	0.000 **	±5.467
				C_W_	0.000 **	
		C_W_	12.807 mM/single-electron Trolox equivalent	Ex_W150_	0.000 **	±1.222
				Ex_W300_	0.000 **	

The multiple pairwise comparisons test and post hoc Bonferroni procedure showed significant differences between the averages for each pair of groups : * means *p* ≤ 0.05, ** means *p* ≤ 0.01. C_PM,_ C_W_—control group, Experimental groups—Ex_PM150_, Ex_PM300_ and Ex_W150_, Ex_W300_ (150 or 300 g of chokeberry pomace additive per each kg of the complete compound feed).

**Table 8:** Analytical composition of applied feed.

Calculated Analyses (kg)	Basic Feed	Chokeberry Pomace
UFV ^1^	0.98	NA
Crude protein (%)	19.79	3.2
PDIA ^2^	13.4	NA
PDIN ^2^	6.8	NA
PDIE ^2^	13.0	NA
Fat (%)	3.23	0.64
Crude fibre (%)	6.87	29.69
Ash (%)	7.64	2.87

^1^ The energy value by INRA system [36]; ^2^ Protein composition value by INRA system [36]; NA—not analysed; UFV—feed unit for maintenance and meat production, PDIA—the dietary protein undegraded in the rumen but truly digestible in the small intestine, PDIN—proteins digestible in the intestine when fermentable nitrogen is limiting, PDIE—proteins digestible in the intestine when rumen fermentable energy are limiting; Vitamins, pro-vitamins, and chemicals in basic feed: Vit A 18,000 j.m.; Vit D3 6050 j.m.; Vit E (DL-Alfa-Tocoferol acetate) 42.0 j.m.; Mixtures of trace elements: Iron (ferrous sulphate monohydrate) 12.5 mg; Selenium (sodium selenite) 0.2 mg; Copper (copper sulphate pentahydrate) 5.0 mg; Manganese (manganese oxide), 27.5 mg; Zinc (zinc oxide) 37.5 mg; Iodine (anhydrous calcium iodate) 0.5 mg; Cobalt (cobalt carbonate monohydrate) 0.1 mg.

**Table 9:** Nonenzymatic antioxidants capacity in all types of feed.

All types of feed	Nonenzymatic Antioxidant Capacity
Chokeberry pomace	974.56 mM/single-electron Trolox equivalent
Basic feed	247.45 mM/single-electron Trolox equivalent
Basic feed + 150 g chokeberry pomace	497.33 mM/single-electron Trolox equivalent
Basic feed + 300 g chokeberry pomace	744.55 mM/single-electron Trolox equivalent
